# Manipulation of Non-canonical NF-κB Signaling by Non-oncogenic Viruses

**DOI:** 10.1007/s00005-018-0522-x

**Published:** 2018-09-08

**Authors:** Justyna Struzik, Lidia Szulc-Dąbrowska

**Affiliations:** Division of Immunology, Department of Preclinical Sciences, Faculty of Veterinary Medicine, Warsaw University of Life Sciences-SGGW, Ciszewskiego 8, 02-786 Warsaw, Poland

**Keywords:** Antiviral immunity, Non-canonical NF-κB signaling, Non-oncogenic viruses

## Abstract

Nuclear factor (NF)-κB is a major regulator of antiviral response. Viral pathogens exploit NF-κB activation pathways to avoid cellular mechanisms that eliminate the infection. Canonical (classical) NF-κB signaling, which regulates innate immune response, cell survival and inflammation, is often manipulated by viral pathogens that can counteract antiviral response. Oncogenic viruses can modulate not only canonical, but also non-canonical (alternative) NF-κB activation pathways. The non-canonical NF-κB signaling is responsible for adaptive immunity and plays a role in lymphoid organogenesis, B cell development, as well as bone metabolism. Thus, non-canonical NF-κB activation has been linked to lymphoid malignancies. However, some data strongly suggest that the non-canonical NF-κB activation pathway may also function in innate immunity and is modulated by certain non-oncogenic viruses. Collectively, these findings show the importance of studying the impact of different groups of viral pathogens on alternative NF-κB activation. This mini-review focuses on the influence of non-oncogenic viruses on the components of non-canonical NF-κB signaling.

## Introduction

The activation of host antiviral defense mechanisms relies on the subset of transcription factors that regulate innate immune response against pathogens, leading to the transcription of genes encoding molecules that help establish antiviral state (Olagnier and Hiscott 2012). Among transcription regulators of innate antiviral immune response, nuclear factor (NF)-κB/p65 and interferon regulatory factor (IRF)3 act as primary drivers of transcription in response to viral infection. These antiviral factors are controlled by host ubiquitin systems and translocate from the cytoplasm to nucleus in response to external stimuli, such as virus infection. Nuclear translocation of antiviral factors serves the subsequent regulation of transcription of genes involved in immunity and inflammation (Freaney et al. 2013; Rajsbaum and García-Sastre 2013).

Proinflammatory cytokines [e.g., tumor necrosis factor (TNF)-α, interleukin (IL)-1β], B and T cell receptors, as well as viral and bacterial components binding Toll-like receptors (TLRs), induce canonical (classical) NF-κB signaling cascades (Dejardin 2006; Espinosa et al. 2011; Lawrence 2009). Upon canonical NF-κB stimulation, TNF receptor (TNFR)-associated factor (TRAF) adaptor proteins undergo ubiquitination. Afterwards, transforming growth factor-β-activated kinase 1 activates the inhibitor κB (IκB) kinase complex (IKK) composed of IKKα, IKKβ and IKKγ/NF-κB essential modulator (NEMO) subunits. IKK, in turn, induces IκB phosphorylation and its subsequent proteasomal degradation, leading to a rapid release of NF-κB dimers, including p65/p50, which then translocate to the nucleus and activate the transcription of immune and inflammatory genes. In general, canonical NF-κB is independent of protein synthesis and has diverse functions (Hayden and Ghosh 2012; Sun 2011).

The non-canonical (alternative) NF-κB activation pathway requires certain TNFR superfamily receptors, such as TNFR2, lymphotoxin-β receptor (LT-βR), B cell activating factor receptor (BAFFR), receptor activator of NF-κB (RANK), CD40, and Fn14. LT-βR is expressed in lymphoid stromal and epithelial cells, and BAFFR—mainly in B cells, whereas CD40 is attributed to different types of cells, such as dendritic cells (DCs), monocytes, B cells, endothelial and epithelial cells, as well as neurons. RANK receptors, in turn, can be found on osteoclast precursors, DCs and activated B cells (Sun 2011, 2012).

**Fig. 1:**
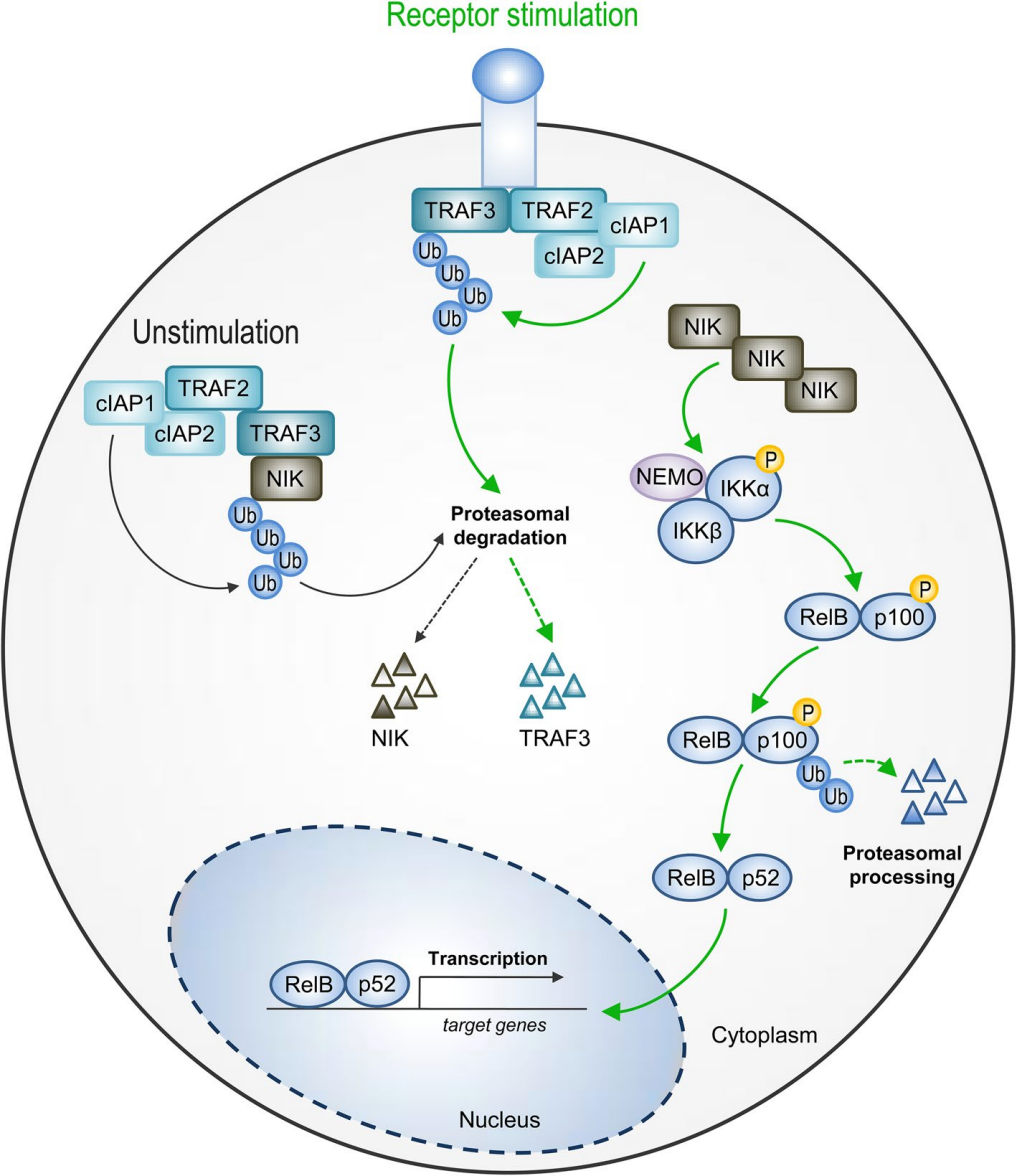
Non-canonical NF-κB activation pathway. In unstimulated cells, NIK kinase is constitutively degraded by cIAP1/2–TRAF2–TRAF3 E3 ubiquitin (Ub) ligase complex. Upon receptor stimulation, TRAF3 undergoes degradation via the activity TRAF2 and cIAP1/2. Thus, NIK is stabilized and accumulates into the cytoplasm. NIK, in turn, activates IKKα kinase. Both NIK and IKKα trigger RelB-associated p100 precursor protein phosphorylation and its subsequent proteasomal processing to active p52 subunit. As a result, RelB/p52 heterodimers translocate to the nucleus to regulate transcription of target genes

Non-canonical NF-κB receptors bind TRAF2 and TRAF3, which are subsequently degraded. TRAF degradation is critical for the activation of NF-κB-inducing kinase (NIK) and IKKα kinase. Importantly, NIK, which links IKKα and p100, is an IKKα activating kinase. Both IKKα and NIK induce selective p100 (Ser866 and Ser870) phosphorylation, which leads to the recruitment of ubiquitin ligase composed of Skp1, Cullin-1 and F-box (Skp–Cullin–F-box, SCF). SCF-mediated processing of RelB-associated NF-κB2 p100 to p52, a central step during non-canonical NF-κB signaling, results predominantly in the activation of p52/RelB complexes (Sun 2011, 2012) (Fig. 1). Unlike the canonical NF-κB activation pathway, the slow and persistent non-canonical NF-κB activation pathway depends on protein synthesis.

Non-canonical NF-κB signaling is responsible for specific functions, including lymphoid organogenesis, DC activation, maturation and survival of B cells, as well as bone metabolism (Biswas and Lewis 2010; Dejardin 2006; Espinosa et al. 2011; Hayden and Ghosh 2012; Hoesel and Schmid 2013; Oeckinghaus and Ghosh 2009; Sun 2011). Deregulation of the non-canonical NF-κB activation results in inflammation, autoimmunity, osteoporosis and lymphoid malignancies (Sun 2011).

Canonical and non-canonical NF-κB activation pathways are connected via many crosstalk mechanisms (Shih et al. 2011). Canonical NF-κB signaling activators, such as TNF-α, lipopolysaccharide (LPS), interferon (IFN)-γ, or 12-*O*-tetradecanoylphorbol-13-acetate (TPA), may stimulate the non-canonical NF-κB activation pathway (Kim et al. 2011). Other studies have demonstrated the involvement of LT-β and LPS in the activation of p100 to p52 functional subunit processing at the ribosome level (Mordmüller et al. 2003).

The common regulation of canonical and non-canonical NF-κB activation is orchestrated by TRAF adaptor molecules that may positively or negatively regulate both signaling pathways. Among these, TRAF3 is critical for virus-induced IRF3-IRF7 activation and controls the non-canonical NF-κB pathway. Under resting conditions, TRAF3 associates with NIK and mediates NIK ubiquitination and proteasomal degradation. Upon stimulation, TRAF3 is degraded via the activity of TRAF2 and the cellular inhibitor of apoptosis (cIAP). Thus, NIK is stabilized (Oeckinghaus et al. 2011). The importance of TRAF3 in non-canonical NF-κB signaling has been demonstrated on TRAF3-deficient mouse embryonic fibroblasts and B cells, which show NIK accumulation together with constitutive p100 processing (He et al. 2006). Similarly, in B cells, TRAF2 or TRAF3 deficiency resulted in NIK accumulation and constitutive activation of the non-canonical NF-κB signaling pathway. TRAF1 may enhance the activity of the cIAP–TRAF2–TRAF3 E3 ubiquitin ligase complex, which drives destruction of NIK. Additionally, ablation of two cIAP1 and cIAP2 proteins, which are functionally redundant, results in NIK stabilization and the activation of the non-canonical NF-κB signaling pathway (Yang and Sun 2015). However, both IKKα and the TRAF family member-associated NF-κB activator-binding kinase 1 phosphorylate NIK leading to the induction of its degradation. As a result, the non-canonical NF-κB signaling is abolished (Vallabhapurapu et al. 2013).

Functional cooperation between canonical and non-canonical NF-κB signaling pathways is mediated by NF-κB dimerization. However, the mechanism of NF-κB subunit dimerization may also mediate negative crosstalk between both pathways. An example of such regulation is the ability of RelA to repress RelB in TNF-α-stimulated cells. As a consequence of RelB binding by RelA, the DNA binding activity of RelB is abrogated. Nevertheless, upon independent activation of RelA and RelB by LT-βR and TNF-α, the synergistic induction of chromatin remodeling within the granulocyte–macrophage colony-stimulating factor promoter can be observed (Sun 2012). Non-canonical NF-κB signaling suppresses the binding of RelA to the *ifnb* gene promoter and appears to regulate histone modification in this promoter (Jin et al. 2014). IKKα may switch off the canonical NF-κB pathway. This strategy leads to the resolution of inflammation and prevents tissue injury. At the same time, IKKα can regulate adaptive immunity via the non-canonical pathway. Therefore, inhibition of IKKα may be utilized as a therapeutic approach (Lawrence 2009). Another NF-κB signaling regulatory strategy is represented by A20, an ubiquitin-editing enzyme, which is involved in switching from canonical to non-canonical NF-κB signaling upon LT-βR stimulation by binding of cIAP1, which results in dissociation of TRAF2/TRAF3 interaction (Yamaguchi et al. 2013). Also, Akt kinase, which activates canonical NF-κB signaling, promotes p100 processing leading to p52 generation (Gustin et al. 2006).

NF-κB is activated by viruses which are linked to malignant cell transformation due to their anti-apoptotic effect on host cells. Pathogens belonging to oncogenic viruses persistently activate the non-canonical NF-κB pathway due to the presence of viral factors that help establish the activated state of NF-κB. A great example of a highly specialized NF-κB activating virus is human immunodeficiency virus type 1 (HIV-1). HIV-1 triggers the two (canonical and non-canonical) NF-κB signaling pathways via the Vpr protein, which enhances the phosphorylation of IKKα/β (Liu et al. 2013). Similarly, the Tax protein expressed by human T-cell leukemia virus type 1 stimulates canonical and non-canonical NF-κB pathways, but requires both IKKα and IKKγ subunits to exert this effect. Kaposi’s sarcoma-associated herpesvirus, in turn, encodes an anti-apoptotic protein, viral FLICE (Caspase-8) inhibitory protein (FLIP), a homolog of the cellular FLIP, which activates canonical and non-canonical NF-κB signaling via IKKγ. Another example of an NF-κB oncogenic activator is latent membrane protein 1 encoded by Epstein–Barr virus, which activates NF-κB signaling via TRAF molecules. Among viral products that activate both non-canonical and canonical NF-κB signaling is oncoprotein Tio encoded by herpesvirus ateles (Sun 2011; Zhao et al. 2015).

**Fig. 2:**
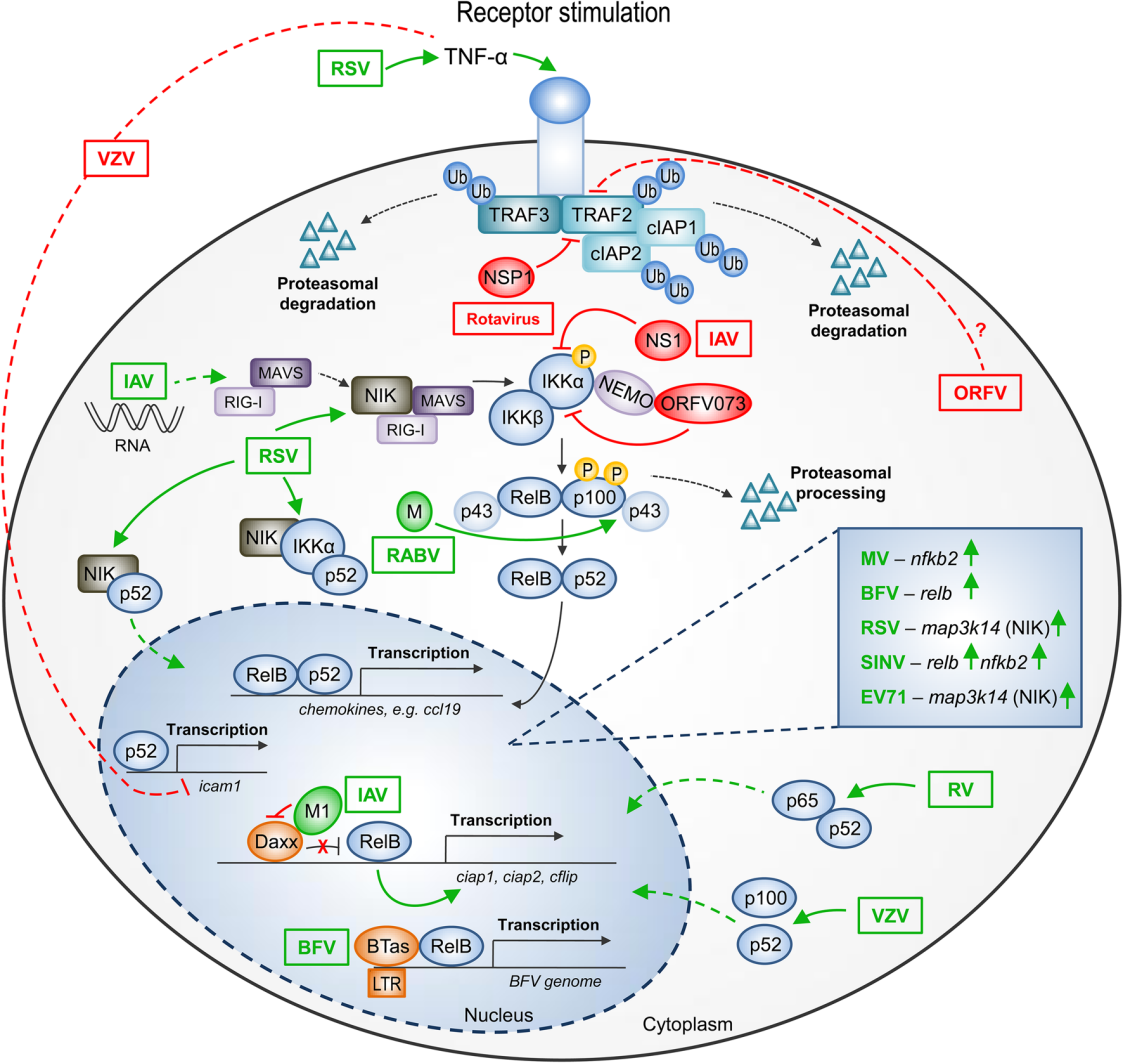
Influence of non-oncogenic viruses on the components of non-canonical NF-κB signaling. A schematic representation of the non-canonical NF-κB signaling pathway and its modulation by non-oncogenic viruses. Activation of the major components of the non-canonical NF-κB pathway by viruses is indicated by pointing green arrows, whereas inhibition—by red blunt arrows. The effects of viral pathogens on the non-canonical NF-κB signaling are described in the text

Although activation of non-canonical NF-κB signaling by certain viruses is attributed to their anti-apoptotic effect and tumorigenesis, other data reveal that the non-canonical NF-κB pathway controls the induction of type I IFNs. This finding demonstrates the unexpected role of the non-canonical NF-κB activation pathway in antiviral innate immunity (Jin et al. 2014). Meanwhile, non-oncogenic viruses (both RNA and DNA) can influence the components of non-canonical NF-κB signaling, which consequently leads to modulation of antiviral immune response (Fig. 2).

## RNA Viruses

### Orthomyxoviridae

Influenza A virus (IAV), a single-stranded (ss), negative sense-(−)RNA orthomyxovirus of *Influenzavirus A* genus, is known for its antigenic drift and is well adapted to its hosts due to the multiple interactions between viral, host and environmental factors (Yoo et al. 2018). The role of NF-κB during IAV infection of upper and lower respiratory tract lung epithelial cells remains unclear. Nevertheless, it is assumed that it is the viral genotype that determines susceptibility to the antiviral functions of NF-κB during IAV infection (Dam et al. 2016).

IAV encodes the non-structural NS1 protein, which antagonizes IFN-mediated antiviral response (Krug 2015). NS1 has been shown to suppress RNA-induced non-canonical NF-κB signaling triggered by the retinoid acid-inducible gene (RIG-I)/mitochondrial antiviral signaling (MAVS) protein. In A549 lung epithelial cells NS1 prevents RIG-I-dependent *chemokine (C–C) ligand (ccl) 19* gene expression, which depends on non-canonical NF-κB activation. As a result, IAV may counteract the recruitment of immune cells to the infected tissue (Rückle et al. 2012). Furthermore, other data show that NS1 encoded by H5N1 and WSN H1N1, a low pathogenic isolate, interacts with IKKα in vitro and in vivo. NS1–IKKα interaction inhibits NIK-induced p100 processing, and, as a consequence, non-canonical NF-κB signaling (Gao et al. 2012).

Halder et al. (2013) showed that RelB transcript level remains unchanged during IAV infection. However, RelB-regulated survival *ciap1, ciap2*, and *cflip* genes undergo activation by IAV matrix protein 1 (M1), which binds death domain-associated protein 6 (Daxx) (Halder et al. 2013). Daxx, in turn, is a transcriptional repressor of RelB, which binds RelB and is involved in epigenetic silencing of RelB-regulated genes. Daxx acts via recruitment of DNA methyltransferase 1 to promoters of target genes and can control genes responsible for apoptosis regulation (Croxton et al. 2006; Puto and Reed 2008). As a result of M1–Daxx interaction, Daxx is not able to bind to *ciap1, ciap2*, and *cflip* gene promoters. Therefore, survival genes are upregulated (Halder et al. 2013).

### Paramyxoviridae

Respiratory syncytial virus (RSV) is a member of the *Pneumovirus* genus and *Pneumovirinae* subfamily of (−)ssRNA paramyxoviruses. In humans, RSV is regarded as a major causative agent of severe lower respiratory tract infection, which leads to lung immune cell infiltration (Canedo-Marroquín et al. 2017).

For in vitro studies of RSV infection, A549 lung-derived human epithelial cell line can be used (Choudhary et al. 2005). In this model of RSV infection, an increase in both NIK mRNA and protein expression, as well as NIK kinase activity were observed. In RSV-infected cells, activation of non-canonical NF-κB signaling precedes stimulation of more influential canonical NF-κB signaling (Choudhary et al. 2005). The induction of non-canonical NF-κB signaling during RSV infection is mediated by TNF-α, which activates NF-κB2 and RelB (Dave et al. 2014). In RSV-infected cells, the formation of the NIK/IKKα/p52 complex and NIK/p52 nuclear translocation can be detected. Additionally, NIK knockdown via siRNA prevents RSV-induced p100 processing in the proteasome, together with the abrogation of activation of NF-κB-regulated genes (Choudhary et al. 2005).

As mentioned above, canonical NF-κB signaling follows the non-canonical pathway during RSV infection. However, RelA, a canonical NF-κB component, is partially activated by RSV via the NIK/IKKα complex. Upon RSV infection, NIK/IKKα interacts with MAVS, an adaptor protein of RIG-I. The NIK/IKKα complex, in turn, triggers RelA release from p100 in the cell cytoplasm. RIG-I silencing leads to the inhibition of basal p100 processing and p100 processing activated by RSV. NIK–IKKα activation may inhibit inflammatory chemokine secretion. These findings lead to the conclusion that NIK–IKKα signaling may be considered a therapeutic target in RSV infections (Liu et al. 2008).

Other studies on RSV infection show the role of NF-κB2 in the induction of antiviral gene expression. Among antiviral cytokines, IL-15 plays a prominent role in host antiviral defense. In *nf-kb2*^−/−^ bone marrow derived macrophages (BMDMs) infected with RSV, induction of IL-15 gene transcription is abrogated. Additionally, BMDM treatment with polyinosinic:polycytidylic acid [poly(I:C)] via TLR3 results in binding of NF-κB2 to the Sp1 promoter to induce transcription, which depends on NF-κB signaling components, including NF-κB2. NF-κB2 p52, in turn, is considered a target for IKKε, thus playing a role in antiviral immunity (Doyle et al. 2013).

Another member of the *Paramyxoviridae* family, the measles virus (MV), belonging to genus *Morbillivirus*, is a pathogen with the only reservoir in humans, which infects different cell types (Da Fontoura Budaszewski and von Messling 2016; Moss 2017). The study of non-canonical NF-κB signaling in MV Edmonston strain-infected human peripheral blood mononuclear cells has led to observations that both mRNA and protein levels of NF-κB2 p52 are upregulated in virus-infected cells. In general, p52/p52 form homodimers, which exert a repressive effect on transcription; however, MV induces upregulation of B cell lymphoma protein-3, which may bind p52 homodimers resulting in the counteraction of transcription repression induced by p52/p52 homodimers (Bolt et al. 2002).

### Picornaviridae

Human enterovirus 71 (EV71) belonging to genus *Enterovirus*, is a positive sense-(+)ssRNA virus within the *Picornaviridae* family and a causative agent of hand, foot, and mouth disease. EV1 infection results in a febrile and highly contagious illness that may lead to neurological complications (Cox et al. 2017; Yi et al. 2017). During the early and late hours post-infection by a rhabdomyosarcoma cell line with EV71, enhanced gene expression of mitogen-activated protein kinase (MAPK) signaling pathway components, such as NIK, a key regulator of non-canonical NF-κB signaling, can be detected. This observation leads to the conclusion that the activation of MAPK signaling upon EV71 infection may be responsible for inflammatory cytokine secretion, as well as apoptosis of EV71-infected cells (Shi et al. 2013).

Another representative of the genus *Enterovirus*, Rhinovirus (RV), which is known as the cause of the mild common cold, can also be detected in patients with pneumonia and other respiratory tract infections (To et al. 2017). RV infection stimulates the activation of NF-κB transcription factors and cytokine production (Zhu et al. 1996, 1997). In particular, in A549 cells infected with the RV14 serotype, the induction of the transcriptional activation of NF-κB family proteins, including p52, but not RelB, can be observed. Therefore, it is likely that p52–p65 heterodimers are activated by RV; yet, transcription of particular genes requires additional cofactors (Zhu et al. 1996).

### Reoviridae

*Rotavirus* is a genus that encompasses double-stranded (ds)RNA viruses within the *Reoviridae* family and is a most common causative agent of severe infantile gastroenteritis (Burnett et al. 2018; Mokomane et al. 2018). The rotavirus genome is composed of 11 segments, which encode six structural and non-structural proteins, whose number depends on the virus strain (Hu et al. 2012). Among rotavirus-encoded non-structural proteins, NSP1 interacts with TRAF2 resulting in its degradation and inhibition of IFN-β-mediated p52 activation. This inhibition of non-canonical NF-κB signaling and cytokine response may be a viral strategy for evading immune effector mechanisms (Bagchi et al. 2013).

### Retroviridae

Bovine foamy virus (BFV), known as bovine syncytial virus, belonging to the genus *Spumavirus* and *Spumaretrovirinae* subfamily of *Retroviridae*, an ss(+)RNA virus that infects its host with no association with clinical outcome (Hechler et al. 2012). BFV utilizes host signaling pathways to enhance its replication in host cells. This ability was demonstrated in vitro by Wang et al. (2010b) who reported that BFV activates non-canonical NF-κB signaling, including IKKα and p100, which undergo processing by NF-κB transactivator BTas. This activation may increase viral transcription (Wang et al. 2010b).

BFV has also been shown to interact with RelB, which can bind to the BFV long terminal repeat (LTR) and interacts with RelB transactivator BTas in vitro and in vivo. In addition, *relb* gene expression is elevated in BFV-infected HeLa cells. Moreover, RelB, which enhances viral transcription in the nucleus, acts as a cotransactivator of BTas. The Rel homology domain is essential for RelB–BTas interaction. This process can be observed due to NF-κB activation via BTas, whose transfection into cells results in increased RelB mRNA expression (Wang et al. 2010a).

### Rhabdoviridae

Rabies virus (RABV), belonging to the *Rhabdoviridae* family and *Lyssavirus* genus, is an (−)ssRNA virus, which evades immune response by blocking cellular pathways activating transcription factors. The matrix (M) protein of RABV interacts with RelAp43, a splicing variant of RelA, and at the same time, a competitor protein of RelA in canonical and non-canonical NF-κB signaling. The non-canonical NF-κB activation pathway is affected by the RABV M protein due to the enhanced interaction between RelAp43 and two components of non-canonical NF-κB signaling: RelB and p100/p52 (Besson et al. 2017; Fooks et al. 2017; Luco et al. 2012).

### Togaviridae

Sindbis virus (SINV), an (+)ssRNA virus belonging to genus *Alphavirus* of the *Togaviridae* family, is transmitted by mosquitoes and may cause persisting musculoskeletal symptoms in humans (Adouchief et al. 2016). Alphaviruses have developed efficient strategies which affect the antiviral response of host cells, including IFN-mediated gene expression (Fros and Pijlman 2016). Nenasheva et al. (2015) studied the response of human embryonic kidney cells to SINV infection. SINV infection resulted in upregulation of *relb* and *nf-kb2* gene expression, and others, such as interferon genes, which may play a role in innate immunity (Nenasheva et al. 2015).

## DNA Viruses

### Herpesviridae

Varicella zoster virus (VZV), also known as human herpesvirus type 3, is a dsDNA virus belonging to genus *Varicellovirus* and the *Alphaherpesvirinae* subfamily of the family *Herpesviridae*. VZV is a causative agent of highly contagious varicella (chickenpox) (Bollaerts et al. 2017; Sauerbrei 2016). In vitro studies on VZV pathogenesis have shown that in VZV-infected melanoma MeWo cell line and MRC5 human lung fibroblast cells, increased levels of nuclear p100 and p52 can be observed. Moreover, the inhibition of TNF-α-induced recruitment of NF-κB, including p52 to the *icam1* promoter during VZV infection has been detected in MeWo cells (El Mjiyad et al. 2007).

### Poxviridae

Among DNA viruses, poxviruses are well-known for modulating host immune response and inhibiting canonical NF-κB signaling at multiple levels (Brady and Bowie 2014). Orf virus (ORFV) belonging to the genus *Parapoxvirus* of the *Chordopoxvirinae* subfamily and *Poxviridae* family of large dsDNA viruses is a pathogen in sheep and goats, as well as a zoonotic agent, which can be used as an immunomodulating drug after inactivation (Bergqvist et al. 2017).

ORFV infection results in orf, a dermal disease manifested by contagious pustular dermatitis (Bergqvist et al. 2017; Spyrou and Valiakos 2015). Among ORFV-encoded immunomodulatory molecules, ORFV073 can inhibit IKK activation early on during the infection and interacts with NEMO, which is also involved in non-canonical NF-κB signaling. Therefore, it is assumed that ORFV may also affect TRAF2, which mediates both canonical and non-canonical NF-κB activation (Khatiwada et al. 2017). TRAF2 is also crucial for IFN-induced non-canonical NF-κB signaling (Yang et al. 2005, 2008). On the other hand, TRAF2 promotes other poxvirus, vaccinia virus replication; therefore, it may act as a proviral factor (Haga et al. 2014). Therefore, the evaluation of the impact of ORFV and other poxviruses on non-canonical NF-κB signaling needs further investigation.

## Conclusions and Perspectives

Viruses are masters of immune response modulation. Therefore, we can assume that there are still plenty of mechanisms for subverting host antiviral immune responses that remain elusive. From the time when particular viruses were linked to apoptosis inhibition and tumorigenesis, research efforts on the role of non-canonical NF-κB signaling in viral infection have been focused on oncogenic viruses. Although several datasets present the involvement of non-canonical NF-κB signaling in non-oncogenic viral infections, the emerging role of non-canonical NF-κB activation in innate immunity suggests that new scope for the study of viral pathogenesis has been unravelled.
